# Characterization of regulatory T cells in urban newborns

**DOI:** 10.1186/1476-7961-7-8

**Published:** 2009-07-08

**Authors:** Ngoc P Ly, Begona Ruiz-Perez, Rachel M McLoughlin, Cynthia M Visness, Paul K Wallace, William W Cruikshank, Arthur O Tzianabos, George T O'Connor, Diane R Gold, James E Gern

**Affiliations:** 1 Pediatric Pulmonary Medicine, University of California San Francisco Children's Hospital and UCSF Medical School, San Francisco, CA, USA; 2 Channing Laboratory, Department of Medicine, Brigham and Women's Hospital, Boston, MA, USA; 3 Rho Federal Systems Division, Inc, Chapel Hill, NC, USA; 4 Roswell Park Cancer Institute, Buffalo, NY, USA; 5 Boston University Medical Center, Boston, MA, USA; 6 University of Wisconsin, Madison, WI, USA

## Abstract

**Background:**

In the United States, asthma prevalence is particularly high among urban children. Although the underlying immune mechanism contributing to asthma has not been identified, having impaired T regulatory (Treg) cells at birth may be a determining factor in urban children. The objective of this study was to compare Treg phenotype and function in cord blood (CB) of newborns to those in peripheral blood (PB) of a subset of participating mothers.

**Methods:**

Treg numbers, expression, and suppressive function were quantified in subjects recruited prenatally from neighborhoods where ≥ 20% of families have incomes below the poverty line. Proportion of Treg cells and expression of naïve (CD45RA) or activated (CD45RO, CD69, and HLA-DR) markers in CD4^+^T cells was measured by flow cytometry. Treg suppressive capacity was determined by quantifying PHA-stimulated lymphocyte proliferation in mononuclear cell samples with and without CD25 depletion.

**Results:**

In an urban cohort of 119 newborns and 82 mothers, we found that newborns had similar number of cells expressing FOXP3 as compared to the mothers but had reduced numbers of CD4^+^CD25^+^bright cells that predominantly expressed the naïve (CD45RA) rather than the activated/memory (CD45RO) phenotype found in the mothers. Additionally, the newborns had reduced mononuclear cell TGF-β production, and reduced Treg suppression of PHA-stimulated lymphocyte proliferation compared to the mothers.

**Conclusion:**

U.S. urban newborns have Treg cells that express FOXP3, albeit with an immature phenotype and function as compared to the mothers. Longitudinal follow-up is needed to delineate Treg cell maturation and subsequent risk for atopic diseases in this urban birth cohort.

## Introduction

The ability of CD4^+^CD25^+ ^T regulatory (Treg) cell to down-regulate immune responses associated with asthma in experimental animal models [1-4] has recently ignited interest in defining the role of Treg cells in allergy and asthma in humans. Most studies on the association between Treg and asthma/allergy have focused on adults [5-8] with allergy or on children [9] with established asthma. Since a majority of cases of asthma are diagnosed in early childhood, [10,11] characterizing Treg phenotype and function in at-risk children prior to the clinical manifestation of asthma may provide a more cohesive understanding of Treg ontogeny and the impact dysregulated Treg have on the development of asthma. Recently, two studies have suggested that Treg function may be impaired among newborns with either a parental [12] or more specifically a maternal [13] history of atopy. While parental atopy/asthma is a risk factor [14-16] for childhood asthma, environmental factors [17,18] also play a significant role in asthma development. In the United States, asthma tends to be more prevalent [19] and severe [20] among urban children as compared to non-urban children.[21] Neonatal and infant Treg phenotype and function, which may influence asthma and allergy development, have not been characterized in an urban birth cohort. In this study we compared Treg numbers, expression, and function in newborns to a subset of mothers participating in the Urban Environment and Childhood Asthma (URECA) study.

## Methods

### Study population

Study subjects included a subset of newborns and mothers from the Boston metropolitan area who participated in the URECA (Urban Environment and Childhood Asthma) Study, a multi-center birth cohort study examining the relationship between immune responses, the environment, and asthma development [22] Subjects were enrolled from February 2005 to March 2007. Inclusion criteria were residence in census tracts with at least 20% of the residents having income below the poverty level; gestational age ≥ 34 weeks; a parental history of atopic disease (asthma, hay fever, or eczema); plan to deliver at the study hospital; maternal ability to speak English or Spanish; and access to a phone. Exclusion criteria were maternal HIV infection at delivery; plans to move out of geographic area during the period of the study; newborn respiratory distress requiring intubation and ventilation for ≥ 4 hours after delivery or supplemental oxygen and/or CPAP for ≥ 4 days; significant congenital anomalies; and immediate postnatal antibiotic treatment for pneumonia. This study was approved by the Institutional Review Boards of Boston University and Brigham and Women's Hospital.

### Demographic, birth, parental conditions, and other variables

Parental demographic and health history were collected by questionnaires. Data on neonatal weight, gestational age, and neonatal intensive care admission were obtained from hospital records.

### Cord and Peripheral Blood Mononuclear Cell Isolation

Umbilical cord blood samples were collected by needle/syringe from the umbilical vein after delivery into heparinized tubes. Peripheral venous blood was obtained from a subset of mothers enrolled in the study at the child's 12-month follow-up visit. At the discretion of the investigator, blood was not obtained from mothers who were acutely ill. All blood samples were processed within 24 hours. Cord and peripheral blood mononuclear cells (MNCs) were isolated by density gradient centrifugation with Ficoll-Hypaque Plus (Amersham Biosciences, UK).

### Depletion of CD25^+ ^T cells

All experiments were performed with fresh, non-cryopreserved cells [22]. The cell sample from each subject was divided into 2 equal aliquots. Depletion of CD25^+ ^T lymphocytes was performed on the first aliquot using MACS columns with a positive CD25^+ ^T-cell selection kit (Miltenyi Biotech Inc., Auburn, CA). The second aliquot was not depleted of CD25^+ ^T cells but was subjected to the same separation process using MACS column with anti-FITC which is an irrelevant antibody (Miltenyi Biotech Inc., Auburn, CA). The CD25^+ ^microbeads removed between 85–95% of CD4^+^CD25^+ ^T cells as analyzed by FACS (data not shown).

### Proliferation assay

Undepleted or CD25^+ ^depleted MNCs (1 × 10^5^/well) were cultured in triplicate in 96 well round-bottom plates containing AIM-V serum-free medium (Invitrogen Corp., Grand Island, NY) alone or with 5 μg/ml PHA added. After 4 days of incubation at 37°C, supernatant for each of the experimental condition was collected and stored at -80°C for future analyses of cytokines. The remaining cell cultures were pulsed for 6 hours with 1 μCi of [^3^H] thymidine/well (NEN™, Life Science Products, Inc., Boston, MA) and proliferation was measured using a β-scintillation counter (Wallac Microbeta Trilux, Perkin Elmer, Waltham, MA). Results were expressed as proliferation index (PI), calculated as ratio of mean counts per minute (cpm) of stimulated over mean cpm of unstimulated cell triplicates.

Regulatory T-cell function has been defined as the ability to suppress lymphocyte proliferation *in vitro*. [23,24] Due to the low numbers of cells available in this study, we adopted a method from Taams *et al *[25] and modified it to indirectly measure suppressive activity of T-regs in participating subjects. The capacity of CD25^+ ^T-cells to suppress proliferation in each subject was determined by comparing lymphoproliferative response of their MNCs to PHA stimulation in a cell sample that was depleted of CD25 to those that were not depleted of the CD25cell population. To establish the effects of CD25 depletion on proliferative activity we also calculated the suppressive index (SI), which is a ratio of PI for CD25depleted to CD25undepleted cell sample.

### TGF-β analysis

TGF-β levels in the cell culture supernatant harvested 4 days after incubation at 37°C were quantified by ELISA using an R&D Systems Duoset (R&D Systems, Inc., Minneapolis, MN) according to the manufacturer's instructions.

### Flow cytometric analysis

For surface staining, aliquots of 2 × 10^6 ^cord or peripheral blood MNCs were washed once in phosphate buffered saline (PBS). The cell pellet was resuspended at approximately 1 × 10^7 ^cells/ml in PBS containing 20 μg/ml mouse IgG (Invitrogen Corporation, Carlsbad, CA) to serve as an Fc receptor block. Tubes were mixed and incubated for 10 min on ice. Subsequently, 50 μl of cells was added to tubes containing cocktails of fluorochrome labeled mAbs. All mAbs were pretitered and used at saturating concentrations. The following mAbs were used in this study (CD3 (clone SK7), CD4 (clone SK3), CD25 (clone 2A3), CD45 (clone 2D1), CD45RA (clone ALB11), CD45RO (clone UCHL.1), CD69 (clone L78), HLA-DR (clone L243) from BD Bioscience (San Jose, CA), FOXP3 (clone 206D) and its isotype control (clone MOPC-21) were purchased from BioLegend (San Diego, CA). The sample tubes were mixed, returned to the ice bath for 30 minutes, and shielded from light to reduce possible photobleaching. After the incubation with mAbs, RBC were lysed with ammonium chloride (0.155 M NH_4_CI, 10 mM KHCO_3_, 0.089 mM EDTA) and washed with PBS before fixing in 2% Ultrapure formaldehyde (Polysciences, Inc., Warrington, PA).

A modification of the surface staining procedure was used for intracellular FOXP3 staining. After the final PBS wash, but before formaldehyde fixation, the cells were resuspended in FOXP3 Fix/Perm buffer (BioLegend, San Diego, CA) and incubated in the dark, at room temperature for 30 minutes. The cells were then washed twice with FOXP3 Perm buffer (BioLegend, San Diego, CA) and resuspended in 50 μl of Perm buffer containing 100 μg/ml human IgG Cohn fraction II and III (Sigma-Aldrich, St. Louis, MO) for 10 minutes before adding the anti-FOXP3 or isotype control mAbs. Cells were incubated for an hour in the dark, washed once with Perm buffer, and then once with PBS before fixing in 2% formaldehyde.

CD4^+^CD25^+^brights were defined by gating on lymphocytes (using forward and side scatter) and CD3^+ ^cells, then using a CD4 versus CD25 histogram a region was created defining the CD4^+^CD25^+ ^(total) and CD4^+^CD25^+^bright cells. The CD4^+^CD25^+^(total) region was defined based on comparison to an isotype control, the CD25^+^bright population was defined in a two step process, first as the population that was brighter than the CD4^-^CD25^+^population and next by their slightly dimmer CD4 intensity as originally defined by Baecher-Allan, C. et al [26]

Stained cells were stored in the dark at 4°C for no longer than 3 days before data acquisition. Samples were analyzed using the FACSCanto cytometer (BD Bioscience, San Jose, CA) running DiVA acquisition software. Excitation signals from FITC (515/30 BP), PE (564/42 BP), PerCP (>670 LP) and PECy7 (750/60 BP) were collected off the solid state 488 nm line and APC (650/20) was collected off the HeNe 633 nm laser line. Cell viability was determined by the Live/Dead fixable green stain according to the manufacturer's recommendations (Invitrogen, Carlsbad, CA). Specimens with viabilities less than 85% were excluded from analysis.

### Statistical Analyses

The Chi-square test was used to compare between-group proportions. The distributions of lymphocyte PI, SI, CD25^+^, CD25^+^bright, FOXP3, and TGF-β expression were skewed; therefore, median levels were presented for each measurement and differences in the levels between CB and PB, and between newborns with and without maternal asthma were examined using nonparametric two-sample Wilcoxon tests. As described above we assessed suppressive activity of CD4^+ ^CD25^+ ^T cells by comparing the PI of samples before and after CD25 depletion, tested using a Signed-rank test for matched comparisons, as well as calculating SI which is a ratio of PI of CD25^+ ^depleted to PI of CD25^+ ^undepleted. The associations between CD25^+^bright, CD25^+^FOXP3+ cell numbers, and SI were determined using Spearman rank correlation. All analyses were performed using SAS, version 9 (SAS Institute, Cary, NC) and the R system for statistical computing [27]

## Results

### Subject characteristics

The subjects in this study consisted of a subset of newborns and mothers enrolled in URECA at the Boston study site. Of the 119 newborns, FACS data characterizing Treg phenotype was available on 114 samples and lymphocyte proliferation data characterizing function was generated on 78 samples. There were no statistical differences in baseline characteristics of newborns with and without proliferation data (Table 1). Although 8 of the infants were admitted to the ICU, none of them were intubated and ventilated. Of the 82 mothers, FACS data was available on 79 and lymphocyte proliferation data was generated on 52 (Table 2). Baseline characteristics were similar among mothers with and without proliferation data except mothers with proliferation data were less likely to have a history of asthma (p < 0.05). Approximately 85% of the mothers (n = 67) had atopy (i.e., asthma, hay fever, or eczema) with 39% of mothers (n = 32) having a maternal history of 2 out of 3 of the diagnoses of eczema, asthma, and hay fever. Of the newborns 80 percent (n = 89) had a maternal history of atopy. FACS analysis and proliferation data were not available for all mother-child pairs because of limitation in cell yields and missed 12-month follow-up visits (for the maternal samples).

**Table 1:** Baseline characteristics of newborns in the URECA study with and without lymphocyte proliferation data.

	**Total** **(N = 119)**	With Data* (N = 78)	Without Data* (N = 41)
		
		**N (%)**	**N (%)**
Sex			
Male	64 (53.8)	44 (56.4)	20 (48.8)
Female	55 (46.2)	34 (43.6)	21 (51.2)
			
Race/ethnicity			
Hispanic	25 (21.0)	16 (20.5)	9 (22.0)
Black	62 (52.1)	43 (55.1)	19 (46.3)
White/Asian/Other	4 (3.4)	2 (2.6)	2 (4.9)
More than one race	24 (20.1)	14 (17.9)	10 (24.4)
Unknown	4 (3.4)	3 (3.9)	1(2.4)
			
NICU admissions	8 (6.7)	4 (5.3)	4 (9.8)
			
Maternal History**			
Eczema	37 (33.0)	23 (31.9)	14 (35.0)
Asthma	59 (53.2)	40 (55.6)	19 (48.7)
Hay fever	51 (46.4)	34 (48.6)	17 (42.5)
			
Paternal History**			
Eczema	22 (17.5)	13 (21.0)	3 (8.3)
Asthma	35 (27.8)	20 (32.3)	9 (25.0)
Hay fever	30 (25.9)	15 (27.8)	12 (34.3)

* No statistically significant difference between newborns with and without lymphocyte proliferation data, p < 0.05.

** Seven of the participants have missing data on maternal and paternal history. Paternal history was reported as unknown in one participant.

**Table 2:** Baseline characteristics of mothers in the URECA study with and without lymphocyte proliferation data

	**Total** **(n = 82)**	**With Data** **(N = 52)**	**Without Data (N = 30)**
		
		**N (%)**	**N (%)**
Race/ethnicity			
Hispanic	24 (29.6)	16 (31.4)	8 (26.7)
Black	40 (49.4)	20 (39.2)	20 (66.7)
White/Asian/Other	7 (8.6)	6 (11.8)	1 (3.3)
More than one race	10 (12.4)	9 (17.7)	1 (3.3)
			
Atopic disease			
Eczema	30 (37.0)	19 (37.3)	11 (36.7)
Asthma*	48 (60.0)	26 (51.0)	22 (75.9)
Hay fever	39 (49.4)	27 (55.1)	12 (40.0)
			
Intake of steroids during pregnancy	18 (22.0)	11 (21.2)	7 (23.3)
		**Mean (SD)**	
Age	26.1 (6.7)	26.9 (7.2)	24.7 (5.7)

* Statistically significant difference between mothers with and without lymphocyte proliferation data, p < 0.05.

### Proportion of CD4^+^CD25^+^bright and CD4^+^CD25^+^FOXP3 T cells in CB and maternal PB

**Figure 1:**
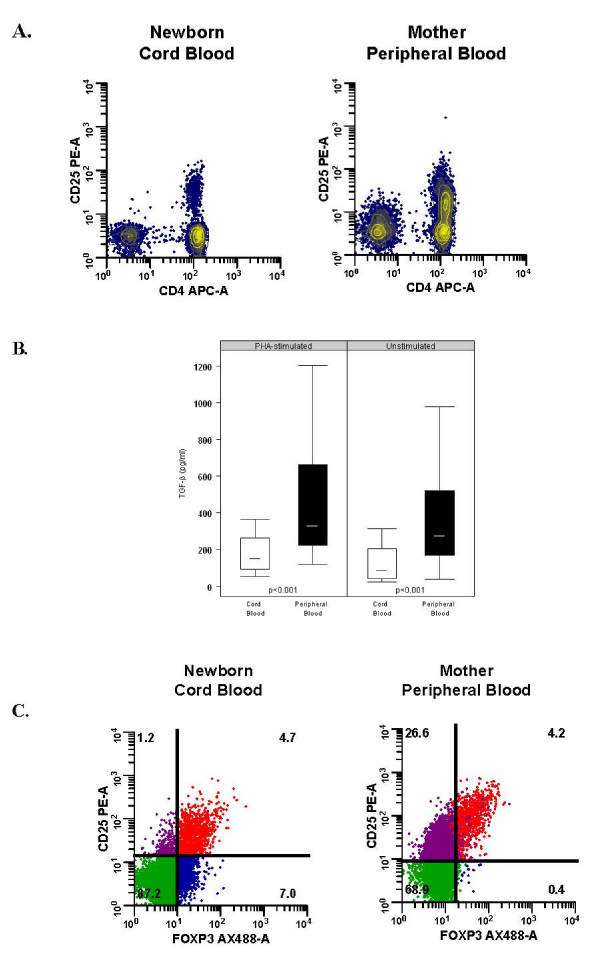
**TGF-β secretion in mononuclear cells and CD25 and Foxp3 expression in CD4^+ ^T-cells of cord blood (CB) and peripheral blood (PB)**. **(A) **Contour plots of CD4 and CD25 expression in unstimulated CB and PB T-cells. Representative examples of one out of 114 CB and 79 PB samples analyzed are shown, illustrating the separation of the CD25^- ^and CD25^+ ^populations in CB CD4^+ ^cells as compared to a broader range CD25 expression in PB CD4^+ ^cells. **(B) **Production of TGF-β cytokines by CB (n = 49) and PB (n = 59) mononuclear cells (MNCs) measured by ELISA in supernatants 4 days after incubation in media (unstimulated) and phytohemagglutinin (PHA). The median is represented by the horizontal bar within the box. The upper and lower boundaries of the box represent the 25^th ^to 75^th ^percentiles of the data, respectively. Observations < 1.5 times the height of the box beyond either quartile are displayed within the whiskers. **(C) **Intracellular expression of Foxp3 in unstimulated samples of CB and PB MNCs analyzed by flow cytometry. The CD4^+ ^cells were gated and analyzed for expression of CD25 and FOXP3. The percentage of CD4^+ ^cells expressing CD25 and FOXP3 is shown in the upper right-hand quadrants. FOXP3 are not distinctly expressed within the CD4^+^CD25^+^bright cell population in CB as compared to PB. Compared to CB, maternal PB had a significant population of CD25^+^FOXP3^- ^cells (upper left-hand quadrants). Results are representative examples of one out of 63 CB and 78 PB samples analyzed.

Considering CD4^+^CD25^+^brightT-cells as marker for regulatory T cells, [26] we compared the proportion of CD4^+^CD25^+ ^and CD4^+^CD25^+^bright T cells in CB and maternal PB (Table 3). We found that CB contained fewer CD4^+^CD25^+ ^and CD4^+^CD25^+^bright T-cells compared to PB. Additionally, we illustrated that while there was a clear separation of CD25^- ^and CD25^+ ^expression on CB CD4^+^cells, there was a broader range of CD25 expression on PB CD4^+ ^cells, including a proportion of CD4 cells that expressed intermediate levels of CD25 (Fig. 1A).

**Table 3:** Proportion of CD4^+^CD25^+ ^cells in cord blood and maternal peripheral blood

	**Cord Blood**			**Maternal Peripheral Blood**			
	N	Median %	Range	N	Median %	Range	Wilcoxon p-value

CD4^+^CD25^+^	114	6.9	0.9–17.7	79	13.3	3.3–38.1	<0.0001
CD4^+^CD25^+^bright	114	1.4	0.2–8.5	79	1.9	0.6–4.5	0.002
CD4^+^CD25^+^FOXP3	63	3.3	0.1–7.8	78	3.1	0.5–6.7	0.71

As TGF-β has previously been shown to up-regulate CD25 expression on CD4^+ ^T-cells in the periphery through induction of FOXP3, [28] we next examined TGF-β production by CB (n = 49) and PB (n = 59) MNCs. Consistent with the finding of a reduced CD25^+ ^cell number in CB, we found lower baseline and PHA-induced TGF-β levels in CB as compared to PB MNCs (Fig. 1B).

FOXP3 transcription factor has been closely associated with Treg cells, (19–21) especially with their development and function [29-31]; therefore, we used intra-cellular staining techniques to analyze FOXP3 expression in the CD25^+ ^population in a subset of participants. We found that the proportion of CD25^+ ^FOXP3^+ ^cells was similar between CB and PB (Table 3); however, the profile of FOXP3 distribution in CD25^+ ^cells differed between CB and PB. For example, in CB, FOXP3 was expressed in CD25 with various levels of expression while in PB, FOXP3 was predominantly expressed in CD25^+^bright cells (Fig. 1C). Moreover, we showed that CD25^+^bright and FOXP3 expression were more tightly correlated in PB (r_s _= 0.56, p < 0.0001) than in CB (r_s _= 0.24; p = 0.05). Compared to CB, maternal PB had a greater proportion of CD25^+^FOXP3^- ^cells that are assumed to represent a higher numbers of activated CD4^+ ^effector cells present in PB (Fig. 1C).

### Comparison of activation marker expression on CB and PB regulatory T-cells

**Figure 2:**
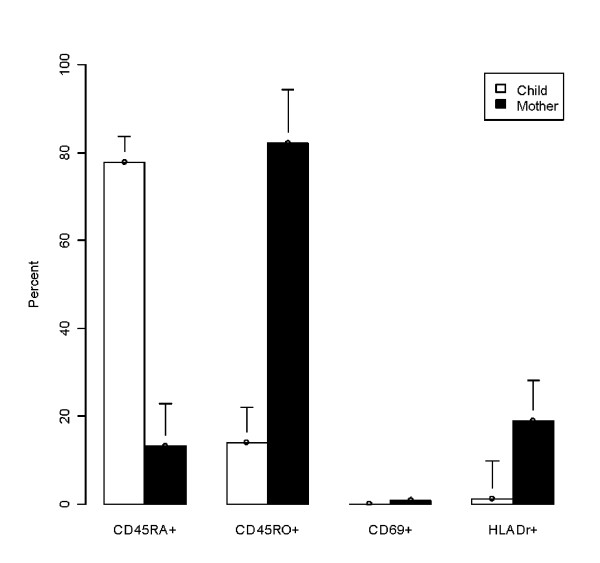
**Comparison of activation markers between cord and peripheral blood CD4^+ ^CD25^+^bright cells**. CD45RO, CD45RA, CD69, and HLA-DR expression on CD4^+ ^CD25^+^bright cells sorted by flow cytometry and expressed in percent. A majority of CB CD4^+^CD25^+^bright cells exhibited a naïve phenotype. In contrast, PB CD4^+^CD25^+^bright exhibited an activated/memory phenotype.

Having identified differences in the numbers of CD25^+^bright cells present in CB and PB, we next sought to establish whether or not these CD25^+^bright cells expressed distinct patterns of differentiation/activation markers (C45RO, CD45RA, HLA-DR, and CD69). CD4^+ ^cells have also been classified as naïve or activated depending on whether they expressed the CD45RA or CD45RO isoform, respectively.[26,32,33] In our samples (Figure 2), CD4^+^CD25^+^bright cells in CB exhibited a naïve phenotype with the majority of cells expressing CD45RA (77.3%) as compared to CD45RO (13.9%). Additionally, only a small percentage of CD25^+^bright cells in CB stained positive for the MHC class II molecule HLA-DR (1.1%) with none of the cells expressing the early activation marker CD69 (0.0%). In contrast, CD25^+^bright cells in maternal PB exhibited an effector memory phenotype, predominantly expressing CD45RO (82.1%), with increased expression of HLA-DR (18.9%) compared to the CB. The differences in CD45RO and HLA-DR expression between CB and PB CD4^+^CD25^+ ^T cell populations were statistically significant (p < 0.02).

### Regulatory T-cell function in cord and maternal peripheral blood MNCs

To determine regulatory T cell function in CB and PB, we analyzed the ability of CD25^+ ^cells to suppress PHA-stimulated lymphocyte proliferation. Depletion of CD25^+ ^cells in CB resulted in little/no change in lymphocyte proliferation (p = 0.56) while depletion of CD25^+^cells in PB resulted in increased lymphocyte proliferation (p = 0.02), suggesting a reduced ability of CD25^+ ^cells in CB to suppress lymphoproliferative response as compared to PB (Table 4). Reduced suppressive function of CD25+cells in newborns compared to their mothers was further illustrated by a lower suppressive index (SI) in CB compared to maternal PB (0.97 vs. 1.22; p < 0.09).

**Table 4:** Lymphocyte proliferation in cord blood and maternal peripheral blood with and without CD25^+ ^depletion

		Proliferation Index (PI)*				
			
		**CD25+ Undepleted**		**CD25+ Depleted**		
	N	Median	Range	Median	Range	Wilcoxon p-value

Cord blood	78	101.8	5.7–776.7	109.9	5.4–943.6	0.56
Maternal Peripheral blood	52	216.7	1.0–713.8	238.3	0.7–798.0	0.02

* Proliferation index (PI) is calculated as ratio of mean counts per minute (cpm) of stimulated over mean cpm of unstimulated cell triplicates.

Next, we examined whether reduced CD4^+^CD25^+ ^number and CD25^+ ^cell suppressive activity in cord blood were associated with having a maternal history of asthma. The proportion of CD4^+^CD25^+ ^(p = 0.20) and CD4^+^CD25^+^bright (p = 0.55) cells were similar between neonates with (n = 57) and without (n = 50) maternal asthma. Interestingly, there was a trend for higher CD25^+^FOXP3^+ ^cell number in neonates with (n = 24) compared those without (n = 34) maternal asthma (median [range] = 2.75 [0.10–7.80] vs. median [range] = 3.85 [1.00–7.30]); p = 0.07). However, reduced CD25^+ ^cell suppressive function was similar between neonates with (n = 40) and without (n = 32) maternal asthma (median [range] = 0.99 [0.11–2.51] vs. median [range] = 0.97 [0.27–4.18]; p = 0.54).

### Association between CD4^+^CD25^+ ^number and suppressive activity

Thus far we have shown that newborns and mothers had different CD4^+^CD25^+ ^cell numbers, phenotype, and function. We next analyzed whether CD4^+^CD25^+ ^cell number is associated with CD25^+ ^cell function. We found no correlation between CD25^+ ^bright cell number and SI levels in CB (r_s _= 0.04: p = 0.725) or in PB (r_s _= -0.14: p = 0.343). Similarly, there was no correlation between CD25^+ ^FOXP3^+ ^cell number and SI level in CB (r_s _= -0.15; p = 0.315) or in PB (r_s _= 0.02; p = 0.905).

## Discussion

The goal of this study was to characterize CD4^+^CD25^+ ^Treg phenotype and function in a U.S. urban birth cohort that is predominantly African American and Latino in ethnicity, and to compare Treg cells from newborns to those of the mothers. In our study, urban newborns had similar number of cells expressing FOXP3 compared to the mothers, but had reduced numbers of CD4^+^CD25^+^bright cells that predominantly expressed the naïve (CD45RA^+^) rather than the activated/memory (CD45RO^+^) phenotype found in the mothers. In addition, the newborns had reduced mononuclear cell TGF-β production, and reduced CD25+ cell suppressive capacity compared to the mothers, regardless of maternal history of asthma. Collectively, these findings suggest that urban newborns have FOXP3 expressing Treg cells with immature phenotype and suppressive capacity compared to the mothers.

Similar phenotypic differences between newborn and adult cells have been reported in studies not specifically selected for urban environment. [26,32,33]The majority of CB CD4^+^CD25^+ ^cells express the naïve T-cell marker CD45RA, while maternal PB CD4^+^CD25^+ ^cells had an activated/memory phenotype and expressed CD45RO. In our study, we also found that maternal CD4^+^CD25^+ ^cells were more likely to express the activation markers HLA-DR and CD69. In contrast to previous findings showing effective suppression of T-cell proliferation by both CB and PB Treg cells, [32,34,35] we found reduced capacity of CD25^+ ^T-cells to suppress PHA-stimulated lymphocyte proliferation in CB as compared to maternal PB. Furthermore, Schaub et al. recently showed reduced number of CD4^+^CD25^+^bright and impaired Treg suppressive function in healthy newborns compared to adults not selected for urban environment. [36] and in offspring of atopic compared to non-atopic mothers [13] TGF-β can induce FOXP3 gene expression and mediate the transition of naive peripheral CD4^+^CD25^-^cells into CD25^+^CD45RB^-/low ^cells with suppressive activity [28] The difference in TGF-β level and FOXP3 distribution in CB and maternal PB may explain the functional differences between the newborns and their mothers. In this study, we compared lymphoproliferative responses in mononuclear cell samples before and after CD25depletion.[25] This method requires relatively few cells, which is an advantage in a large clinical study with limited cell numbers. While CD25 is an imperfect marker of Treg cells, the consistent observation that CD25 depletion resulted in increased lymphoproliferative responses to PHA in maternal PB compared to CB suggests that were are depleting a regulatory cell population.

In our cohort, neither CD4^+^CD25^+^bright nor CD4^+^CD25^+^FOXP3^+ ^cell numbers were associated with CD25^+ ^cell function in CB or maternal PB. The German study, [13] similarly did not find significant association between CD25^+^FOXP3^+ ^cell number and Treg function. Although, FOXP3 transcription factor plays a critical role in Treg development and function, [29-31] FOXP3 is also expressed by non-regulatory CD4^+ ^effector cells upon activation [37,38] Compared to their mothers, newborns had reduced CD25^+ ^cell function despite having similar proportion of cells expressing FOXP3^+^. Furthermore, while there was a trend for higher CD25^+^FOXP3^+ ^cell number in neonates with maternal asthma, CD25^+ ^cell suppressive capacity was similarly reduced in neonates with and without maternal asthma. Further follow-up of these urban neonates is important to determine whether reduced suppressive capacity of Treg cells at birth predicts or predisposes them to asthma and other atopic diseases.

## Conclusion

In conclusion, U.S. urban newborns have Treg cells that express FOXP3, albeit with an immature phenotype and function as compared to the mothers. Longitudinal follow-up is needed to delineate Treg cell maturation and subsequent risk for atopic diseases in this urban birth cohort.

## Abbreviations

Treg: T regulatory cell; MNCs: mononuclear cells; CB: cord blood; PB: peripheral blood; PHA: phytohemagluttinin; cpm: count per minute; PI: proliferation index; SI: suppressive index; mAbs: monoclonal antibodies; FOXP3: foxhead/winged helix transcription factor; URECA: Urban Environment and Childhood Asthma; CPAP: continuous positive airway pressure.

## Competing interests

The authors declare that they have no competing interests.

## Authors' contributions

NPL conducted the data analysis and wrote the manuscript. BRP performed the proliferation studies and participated in data analysis. RMM, CMV, and AOT assisted and participated in data analysis. PKW supervised the flow cytometry studies and participated in data analysis. WWC and DRG participated in study design and supervised the data analysis. GTO supervised patient recruitment for the study and obtained funding. JEG participated in study design, data analysis, and obtained funding. All of the authors participated in drafting the manuscript and approved its final version.
